# In Vitro Anti-Inflammatory Properties of Selected Green Leafy Vegetables

**DOI:** 10.3390/biomedicines6040107

**Published:** 2018-11-19

**Authors:** K. D. P. P. Gunathilake, K. K. D. S. Ranaweera, H. P. Vasantha Rupasinghe

**Affiliations:** 1 Department of Food Science & Technology, Faculty of Livestock, Fisheries & Nutrition, Wayamba University of Sri Lanka, Makandura, Gonawila 60170, Sri Lanka; 2 Department of Food Science and Technology, Faculty of Applied Sciences, University of Sri Jayewardenepura, Gangodawila, Nugegoda 10250, Sri Lanka; sranaweera@sjp.ac.lk; 3 Department of Plant, Food, and Environmental Sciences, Faculty of Agriculture, Dalhousie University, Truro, NS B2N 5E3, Canada; vrupasinghe@dal.ca

**Keywords:** leafy vegetables, anti-inflammatory activity, methanolic extracts, lipoxygenase inhibition, hemolysis inhibition

## Abstract

The study investigated the anti-inflammatory activity of the hydro methanolic extract of six leafy vegetables, namely *Cassia auriculata*, *Passiflora edulis*, *Sesbania grandiflora*, *Olax zeylanica*, *Gymnema lactiferum*, and *Centella asiatica*. The anti-inflammatory activity of methanolic extracts of leafy vegetables was evaluated using four in vitro-based assays: hemolysis inhibition, proteinase inhibition, protein denaturation inhibition, and lipoxygenase inhibition. Results showed that the percent inhibition of hemolysis from these leaf extracts (25–100 µg/mL dry weight basis (DW)) was within the range from 5.4% to 14.9%, and the leaves of *P. edulis* and *O. zeylanica* showed a significantly higher (*p* < 0.05) inhibition levels. Percent inhibition of protein denaturation of these leafy types was within the range of 36.0–61.0%, and the leaf extract of *C. auriculata* has exhibited a significantly higher (*p* < 0.05) inhibition level. Proteinase inhibitory activity of these leaf extracts was within the range of 20.2–25.9%. The lipoxygenase inhibition was within the range of 3.7–36.0%, and the leaf extract of *G. lactiferum* showed an improved ability to inhibit lipoxygenase activity. In conclusion, results revealed that all the studied leaves possess anti-inflammatory properties at different levels, and this could be due to the differences in the composition and concentration of bioactive compounds.

## 1. Introduction

Inflammation is generally referred to as a complex biological response of vascular tissues to harmful stimuli. As well, inflammation is associated with pain, and it involves in an increase of protein denaturation, an increase of vascular permeability, and membrane alteration, among others [1]. Inflammation is also described as the body response to inactivate or eliminate the invading stimuli or organisms, to remove the irritants and set the stage for tissue repair, and the process is accelerated by the release of chemical mediators from injured cells or tissues and migrating cells [2]. The migration of leukocytes from the venous systems to the site of damage, and the release of cytokines, are known to play a crucial role in the inflammatory response [3]. These chemicals cause widening of blood capillaries (vasodilation) and the permeability of the capillaries. This will lead to increased blood flow to the injured site [3]. Inflammation can be classified as either acute or chronic. Acute inflammation is the initial response of the body to harmful stimuli, and is achieved by the progressive movement of plasma and leukocyte-like constituents from the blood, into the injured tissues/locations. Chronic inflammation leads to a progressive shift in the type of cells present at the site of inflammation, and is characterized by simultaneous breakdown and healing of the tissue from the inflammatory process [4]. Non-steroidal anti-inflammatory drugs (NSAID) are commonly used for the management of inflammatory conditions. However, these drugs have several adverse side effects, especially gastric irritation, leading to the formation of gastric ulcers. Therefore, the search for natural sources and phytochemicals with anti-inflammatory activity has greatly increased in recent years. 

Various epidemiological studies provide convincing evidence that natural dietary constituents, such as polyphenols and flavonoids, that humans consume as food, possess many biological activities [5,6]. Further, several epidemiological studies also indicated that the incidence of chronic diseases, such as cancer, cardiovascular diseases, and inflammation, is inversely correlated with the consumption of fruits and vegetables rich in polyphenols, such as flavonoids [7]. Green leafy vegetables are the vital constituent in any balanced diet, and they are rich in polyphenols and antioxidant vitamins. Leafy vegetables, such as *Cassia auriculata*, *Passiflora edulis*, *Gymnema lactiferum*, *Sesbania grandiflora*, and *Olax zeylanica*, are some of the leafy vegetables rich in polyphenols, carotenoids, and antioxidant activities [8,9]. Further, *Centella asiatica* is one of the leafy vegetables that possess anti-inflammatory properties [10]. Though these leafy vegetables have been studied for their antioxidant activities, studies on evaluation of their anti-inflammatory properties have not been reported extensively. Therefore, the present study was conducted to determine the anti-inflammatory activity of selected leafy vegetables using several in vitro bioassays, such as inhibition of albumin denaturation, antiproteinase activity, membrane stabilization, and anti-lipoxygenase activity.

## 2. Materials and Methods

### 2.1. Materials

Fresh green leafy vegetable samples, *Passiflora edulis*, *Olax zeylanica*, *Gymnema lactiferum*, *Sesbania grandiflora*, *Centella asiatica*, and *Cassia auriculata* L. were gathered from the Negombo and Makandura areas in Sri Lanka, and they were cleaned and freeze-dried. A sample from each leafy type were deposited in a herbarium (Herbarium 2014-1) at the Department of Food Science and Technology, Faculty of Livestock, Fisheries & Nutrition, Wayamba University of Sri Lanka.

### 2.2. Reagents

Bovine serum albumin, trypsin, Tris-HCl, perchloric acid, casein, lipoxygenase, linoleic acid, lutein, rutin, β-carotene, and methanol, were obtained from Sigma Aldrich, St. Louis, MO, USA, through Analytical Instrument Pvt Ltd., Colombo, Sri Lanka. All other chemicals used were of analytical grade.

### 2.3. Preparation of Crude Extracts

One gram of freeze-dried sample was mixed with 20 mL of methanol (80% *v*/*v*) and vortexed at high speed for about five minutes, and then centrifuged (Hettich, EBA 20) for 10 min at 4500 rpm, and the supernatants were collected. Then, the extracts were filtered through a filter paper (WhatmanNo.42), and then the residue that remained was re-extracted with 80% methanol with the same procedure, and the supernatants obtained were combined with those from the first extraction. The solvent in the combined mixture was evaporated in a rotary evaporator (HAHNVAPOR, Model HS-2005 V, HAHNSHIN Scientific, Seoul, South Korea) at 40 °C. The prepared concentrated extracts of leafy vegetables were dried at 40 °C for 12 h in an oven, and then dried extracts were stored at −18 °C in air-tight screw-capped glass vials, until used for the anti-inflammatory bioassays, within one week. The extracts collected were dissolved in methanol to obtain a concentration of 3 mg/mL for each assay.

### 2.4. Membrane Lysis Assay

#### 2.4.1. Preparation of Erythrocyte Suspension

Erythrocyte suspension was prepared according to the method described in Shin de et al. [11], with some modifications. Whole human blood was collected from a healthy human subject. The blood was centrifuged at 3000 rpm for 5 min in heparinized centrifuge tubes, and washed three times with equal volume of normal saline (0.9% NaCl). After the centrifugation, the blood volume was measured and reconstituted as a 10% (*v*/*v*) suspension with isotonic buffer solution (10 mM sodium phosphate buffer pH 7.4). Composition of the buffer solution (g/L) used was NaH_2_PO_4_ (0.2), Na_2_HPO_4_ (1.15), and NaCl (9.0).

#### 2.4.2. Heat-Induced Hemolysis

This test was carried out as described by Okoli et al. [12], with some modifications as described in Gunathilake et al. [13]. Briefly, 0.05 mL of blood cell suspension and 0.05 mL of hydromethanolic extracts of leaves were mixed with 2.95 mL phosphate buffer (pH 7.4). The mixture was incubated at 54 °C for 20 min in a shaking water bath. After the incubation, the mixture was centrifuged (2500 rpm for 3 min), and the absorbance of the supernatant was measured at 540 nm using a UV/VIS spectrometer (Optima, SP-3000, Tokyo, Japan). Phosphate buffer solution was used as a control for the experiment.

The level of hemolysis was calculated using the following equation based on the Okoli et al. [12]:

% inhibition of hemolysis = 100 × (1 − A2/A1),(1)

where A1 = absorption of the control, and A2 = absorption of test sample mixture.

#### 2.4.3. Effect on Protein Denaturation

Protein denaturation assay was done according to the method described by Gambhire et al. [14], with some modifications as described in Gunathilake et al. [13]. The reaction mixture (5 mL) consisted of 0.2 mL of 1% bovine albumin, 4.78 mL of phosphate buffered saline (PBS, pH 6.4), and 0.02 mL of extract, and the mixture was mixed, and was incubated in a water bath (37 °C) for 15 min, and then the reaction mixture was heated at 70 °C for 5 min. After cooling, the turbidity was measured at 660 nm using a UV/VIS spectrometer (Optima, SP-3000, Tokyo, Japan). Phosphate buffer solution was used as the control. The percentage inhibition of protein denaturation was calculated by using the following formula:

% inhibition of denaturation = 100 × (1 − A2/A1),(2)

where A1 = absorption of the control sample, and A2 = absorption of the test sample.

#### 2.4.4. Proteinase Inhibitory Activity

Proteinase inhibitory activity of the leaf extracts was performed according to the method of Sakat et al. [15], which is modified by Gunathilake et al. [13]. Briefly, the reaction solution (2 mL) consisted of 0.06 mg trypsin, 1 mL of 20 mM Tris-HCl buffer (pH 7.4), and 1 mL test sample (0.02 mL extract 0.980 mL methanol). The solution was incubated (37 °C for 5 min), and then 1 mL of 0.8% (*w*/*v*) casein was added, and the mixture was further incubated for an additional 20 min. At the end of incubation, 2 mL of 70% perchloric acid was added to terminate the reaction. The mixture was centrifuged, and the absorbance of the supernatant was measured at 210 nm against buffer as the blank. Phosphate buffer solution was used as the control. The percentage inhibition of protein denaturation was calculated by using the following formula:

% inhibition of denaturation = 100 × (1 − A2/A1),(3)

where A1 = absorption of the control sample, and A2 = absorption of the test sample.

### 2.5. Lipoxygenase Inhibition Assay

Lipoxygenase inhibition activity of the extracts of leafy vegetables was assayed according to the method of Wu [16], with some modifications as described in Gunathilake et al. [13]. Briefly, a mixture of a solution of sodium borate buffer (1 mL, 0.1 M, pH 8.8) and lipoxygenase (10 μL, final concentration 8000 U/mL) was incubated with 10 mL leaf extract in a 1 mL cuvette at room temperature (30 ± 2 °C) for 5 min. The reaction was initiated by the addition of 10 µL linoleic acid substrate (10 mmol). The absorbance of the reaction solution was measured at 234 nm using a UV/VIS spectrometer (Optima, SP-3000, Tokyo, Japan). Phosphate buffer solution was used as the control, and the percentage inhibition of lipoxygenase was calculated using the following equation:

% inhibition = 100 × (absorbance of the control − absorbance of the sample)/absorbance of the control(4)

### 2.6. Determination of Polyphenols, Flavonoids, and Carotenoids

Polyphenol contents were measured as described by Gunathilake et al. [17], carotenoid contents were measured as described in Gunathilake et al. [18,19], and flavonoid contents using Gunathilake et al. [20]. These data were used for the correlation studies with anti-inflammatory properties.

### 2.7. Statistical Analysis

All data are presented as the mean ± standard deviation for the all in vitro assays tested, and each analysis was done in triplicate. One-way analysis of variance (ANOVA) was performed using MINITAB 15 software and Pearson’s correlation coefficient (*r*) with the level of significance (*p ≤* 0.05) (2-tailed) for flavonoids, carotenoids, and polyphenols versus anti-inflammatory assays.

## 3. Results

### 3.1. Effect on Hemolysis

**Figure 1:**
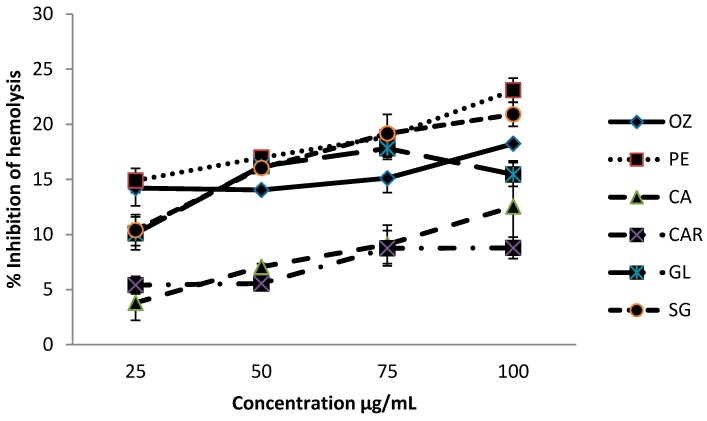
Effect of six green leafy vegetables on inhibition of hemolysis. Values represent means of triplicate readings. CAR—*C. auriculata*; GL—*G. lactiferum*; OZ—*O. zeylanica*; PE—*P. edulis*; CA—*C. asiatica*; SG—*S. grandiflora*. Data are presented as the means ± standard deviations of three replicate determinations.

The percent inhibition of heat-induced hemolysis of red blood cells at different concentrations of each leafy vegetable (DW), in the range of 25–100 µg/mL, is shown in Figure 1.

Methanolic extracts of leafy vegetables were able to inhibit hemolysis in a concentration-dependent manner. Inhibition % of hemolysis from these leaf extracts were within the range from 3.8% to 23.1%, at the concentrations of 25–100 µg/mL. Leaves of *Passiflora edulis* and *O. zeylanica* showed significantly higher (*p* < 0.05) levels of hemolysis inhibition compared to other leafy types studied, and *C. asiatica* showed the least inhibition levels among the six leaves. The order of the inhibition % of extracts of leaves varieties was *P. edulis* > *O. zeylanica* > *C. auriculata* > *G. lactiferum* > *S. grandiflora* > *C. asiatica*. 

### 3.2. Effect of Protein Denaturation

**Figure 2:**
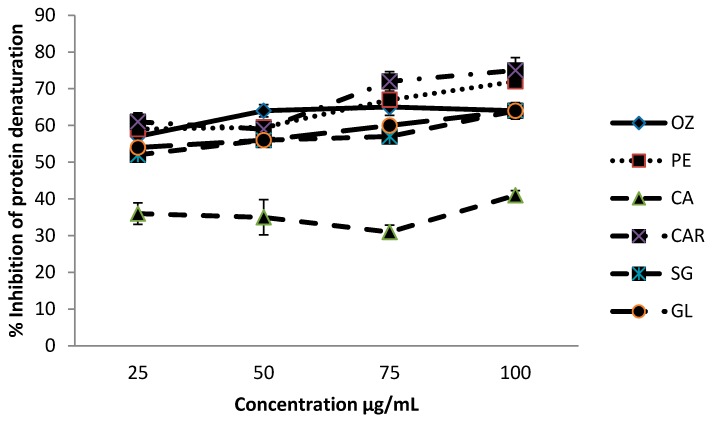
Effects of six green leafy vegetables on protein denaturation. Values represent means of triplicate readings. CAR—*C. auriculata*; GL—*G. lactiferum*; OZ—*O. zeylanica*; PE—*P. edulis*; CA—*C. asiatica*; SG—*S. grandiflora*. Data are presented as the means ± standard deviations of three replicate determinations.

Methanolic extracts of leafy vegetables were able to inhibit protein denaturation in a concentration-dependent manner, and the inhibitory effect of different leafy vegetables at different concentrations (25–100 µg/mL) on protein denaturation is shown in Figure 2. 

Inhibition % of protein denaturation of these leafy vegetables was within the range from 36.0% to 75.0% at the concentration range of 25–100 µg/mL. Leaves of *C. auriculata* exhibited a significantly higher (*p* < 0.05) level of inhibition compared to other leafy types studied, whereas leaves of *C. asiatica* showed the lowest inhibition levels. The order of the inhibition of the extracts of leaves varieties was *C. auriculata* > *P. edulis* > *O. zeylanica* > *G. lactiferum* > *S. grandiflora* > *C. asiatica*.

### 3.3. Proteinase Inhibitory Activities

**Figure 3:**
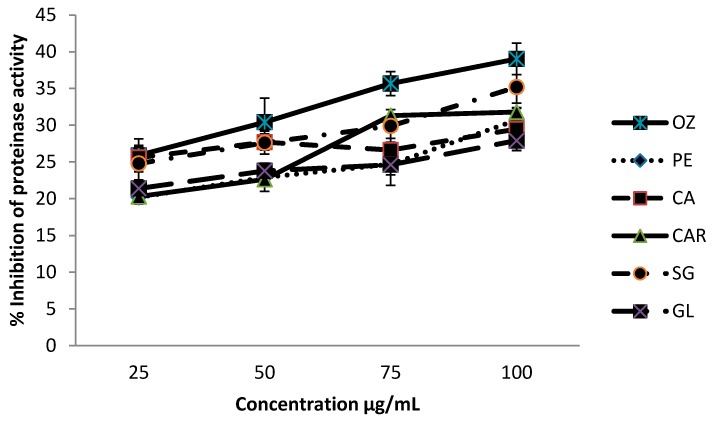
Proteinase inhibitory activity of green leafy vegetables. Values represent means of triplicate readings. CAR—*C. auriculata*; GL—*G. lactiferum*; OZ—*O. zeylanica*; PE—*P. edulis*; CA—*C. asiatica*; SG—*S. grandiflora*. Data are presented as the means ± standard deviations of three replicate determinations.

Proteinase inhibitory activity of different leafy vegetables is shown in Figure 3, and the inhibition levels were within the range of 20.2–39.0%. Leaves of *O. zeylanica* and *S. grandiflora* have shown significantly higher (*p* < 0.05) proteinase inhibition level compared with other leafy types.

### 3.4. Lipoxygenase Inhibition Activity

**Figure 4:**
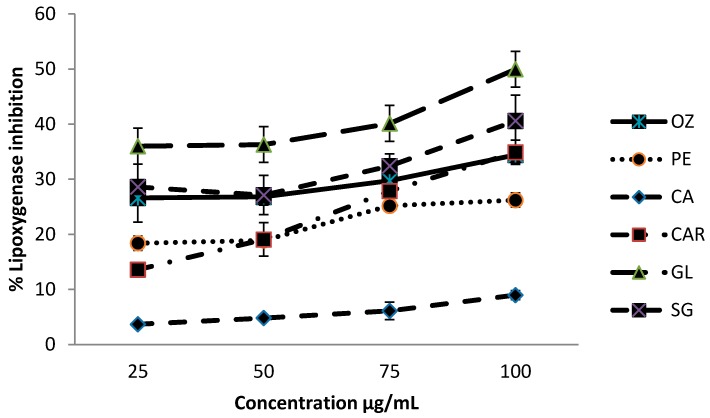
Lipoxygenase inhibitory activity of green leafy vegetables. Values represent means of triplicate readings. CAR—*C. auriculata*; GL—*G. lactiferum*; OZ—*O. zeylanica*; PE—*P. edulis*; CA—*C. asiatica*; SG—*S. grandiflora*. Data are presented as the means ± standard deviations of three replicate determinations.

Results for lipoxygenase inhibitory activity of different leafy vegetables are shown graphically in Figure 4. 

Inhibition levels were within the range of 3.7–50.0% within the concentrations of 25–100 µg/mL. The leaves of *G. lactiferum* showed an improved ability to inhibit lipoxygenase activity (about 50.0%) at 100 µg/mL concentration, whereas *C. asiatica* has shown the least inhibitory activity (9.0%) at the same concentration, among the leafy vegetables studied. The order of the lipoxygenase inhibitory activity of the leaf varieties was *G. lactiferum* > *C. auriculata* > *S. grandiflora* > *P.edulis* > *O. zeylanica* > *C. asiatica*.

The results of this study also showed that there was a significant correlation between the studied anti-inflammatory properties (% inhibition protein denaturation, hemolysis, lipoxygenase activity, and proteinase activity) and their estimated total polyphenol, flavonoid, and carotenoid contents (Table 1). These correlation studies indicate that the evaluated anti-inflammatory properties may be related to the presence of antioxidant bioactives, such as polyphenols, flavonoids, and carotenoids.

## 4. Discussion

Cellular infiltration, due to the pivotal role played by leukocytes, is an important aspect of an inflammatory response [21]. During inflammation, as part of their defensive roles, leukocytes release their lysosomal enzymes, including proteases, causing further tissue damage and subsequent inflammation [12]. Damage to cell membranes will further make the cell more susceptible to secondary damage by means of free radical-induced lipid peroxidation [21]. Regulation of the volume and water content of cells may occur, through membrane proteins, by controlling the movement of sodium and potassium ions and damage to the membrane will affect this function [12]. As the red blood cell membrane is similar to that of lysosomal membrane, inhibition of red blood cell hemolysis may provide insights into the inflammatory process [21]. Stabilization of these cell membranes may retard or inhibit the lysis and subsequent release of the cytoplasmic contents which, in turn, minimize the tissue damage and, hence, the inflammatory response [12]. Therefore, substances that contribute significant protection of cell membrane against injurious substances are important in the event of inhibiting the progression of inflammation. 

In a previous study, it was reported that the aqueous extract of *P. edulis* leaves, and its derived fractions, slow down the inflammation-related responses induced by carrageenan and histamine in the mouse air-pouch model. Furthermore, these extracts inhibit the production of a variety of pro-inflammatory cytokines, mediators, and enzymes at inflammatory sites [22]. However, there is no report available for the anti-inflammatory properties of *O. zeylanica* leaves. In an in vitro study on the effect of *Fagra zanthoxiloides*, *Olax subscorpioidea*, and *Tetrapleura tetraptera*, on membrane stabilization, showed that they inhibited the heat- and hypotonicity-induced lysis of red blood cells [23]. In another study, it was reported that *Albuca setosa* aqueous extract, at the concentration 125–500 μg/mL, may protect the lysis of erythrocyte membrane induced by heat solution [21]. However, the precise mechanism of membrane stabilization is not known, although the interaction of components in the extract with membrane constituents seems most probable. Aitadafoun and colleagues [24] have indicated some plant extracts which show membrane stabilizing properties, and they possess interfering activity with the early phase of the inflammatory mediators’ release, namely, the prevention of phospholipases release that trigger the formation of inflammatory mediators. Further, it is possible that plant extracts may affect the ratio of surface area/volume of the cells by an expansion of membrane, or the shrinkage of cells and interaction with membrane proteins [11].

Denaturation of protein molecules is well documented in the literature, and it is due to an inflammation process in conditions like arthritis [21]. One of the main mechanisms of action of NSAIDs is the protection against protein denaturation as mentioned by Mizushima [25]. Inhibition of protein denaturation may play an important role in the antirheumatic activity of NSAIDs [21]. Previously, the effect of different plant parts on protein denaturation have been evaluated by many scientists, for example, *Semecarpus anacardium* bark on bovine albumin [26], an ethanolic extract of *Wedelia trilobata*on bovine albumin [27], *Albucas etosa*on egg albumen [21], etc. The ability of studied leaf extracts to prevent thermal and hypotonic protein denaturation maybe responsible for their anti-inflammatory properties. Further, various plant extracts have shown their protein denaturation ability, as mentioned earlier. However, the actual mechanism of this membrane stabilization is yet to be investigated further. It has been proposed that the extract might inhibit the release of the lysosomal constituents of neutrophils at the site of inflammation [27]. Lysosomal constituents are bactericidal enzymes and proteinases which, upon extracellular release, cause further tissue inflammation and damage [28].

Proteinases have been associated with arthritic reactions. Neutrophils, in their lysosomal granules, carry many serine proteinases [27]. Proteinases of leukocytes play a significant role in the development of tissue damage during inflammatory processes. According to Das and Chatterjee [29], a significant level of protection was provided by proteinase inhibitors. Various recent studies have shown that many flavonoids contributed significantly to the antioxidant and anti-inflammatory activities of many plants. Therefore, the presence of bioactives present in these leaves may contribute to their anti-inflammatory activity. Our previous studies have shown that these leafy vegetables are rich in polyphenols, flavonoids, and carotenoids [9]. According to our previous study [9], total flavonoid and polyphenolic contents of soluble and bound phenolic fractions of leafy vegetables were within the range of 77.85–325.25 and 33.17–62.30 mmol rutin equivalents/g DW, respectively, and the β-carotene and lutein contents of these studied leafy types were 0.15–0.44 and 0.24–0.77 g/kg DW, respectively.

In many previous studies, methanolic extracts of *Semecarpus anacardium* bark [26], and an ethanolic extract of *Wedelia trilobata* [27] have exhibited significant antiproteinase (trypsin) activity in a dose-dependent manner.

Lipoxygenases are the key enzymes in the biosynthesis of leukotrienes. Leukotrienes play an important role in several inflammatory diseases, such as arthritis, asthma, cancer, and allergic diseases [30]. The mechanism of anti-inflammation may involve a series of events in which the metabolism of arachidonic acid plays an important role [31]. In this process, arachidonic acid is cleaved from the membrane phospholipids upon appropriate stimulation of neutrophils, and can be converted to leukotrienes and prostaglandins through lipoxygenase and cyclooxygenase pathways, respectively [31]. Lipoxygenase catalyzes deoxygenation of polyunsaturated fatty acids to produce *cis*, *trans*-conjugated diene hydroperoxides, such as leukotrienes, which are essential mediators in a variety of inflammatory events [32]. Previous studies have shown that some herbs also have high lipoxygenase inhibitory activity, such as *Leptadenia pyrotechnica* [32] and *Mahonia aquifolium* [30]. These results suggest that *G. lactiferum* has a potentially high anti-inflammatory effect compared with other leafy vegetables studied, which might be related to the polyphenol content and antioxidant property of the extract. Previous studies have shown that polyphenols may block or interfere with the cascade process of arachidonic acid metabolism by inhibiting lipoxygenase activity and, also, they may serve as scavengers of various reactive free radicals which are produced during arachidonic acid metabolism [33]. However, according to Gunathilake et al. [13], most of these anti-inflammatory properties changed according to the type of cooking and the type of leaves.

## 5. Conclusions

In conclusion, results indicate that the hydromethanol extracts of leaves of *C. auriculata*, *P. edulis*, *G. lactiferum*, *S. grandiflora*, *O. zeylanica*, and *C. asiatica* possess anti-inflammatory properties at varying levels. Leaves of *O. zeylanica* and *P. edulis* showed higher hemolysis inhibition activity. Leaves of *C. auriculata* possess higher protein denaturation inhibition properties whereas *G. lactiferum* showed higher lipoxygenase inhibition ability. Leaves of *O. zeylanica*, *S. grandiflora*, and *C. asiatica* exhibited good proteinase inhibition properties. Pearson’s correlation studies showed that there were significant correlations between estimated bioactives and anti-inflammatory properties. Results indicate that these anti-inflammatory activities may be due to the occurrence of bioactive compounds, such as polyphenols, flavonoids, and carotenoids in these leafy types.

## Figures and Tables

**Table 1:** Pearson’s correlation coefficients (*r*) with the level of significance (*p ≤* 0.05) (2-tailed) for flavonoids, carotenoids, and polyphenols, versus anti-inflammatory assays of selected leaf extracts.

Correlation	*r*	*p*
Phenolics versus protein denaturation	0.741	0.001
Phenolics versus hemolysis	0.731	0.001
Phenolics versus lipoxygenase activity	0.531	0.024
Phenolics versus proteinase activity	0.903	0.001
Flavonoids versus protein denaturation	0.842	0.000
Flavonoids versus hemolysis	0.454	0.054
Flavonoids versus lipoxygenase activity	0.388	0.001
Flavonoids versus proteinase activity	0.712	0.001
Carotenoids versus protein denaturation	0.735	0.001
Carotenoids versus hemolysis	0.387	0.112
Carotenoids versus lipoxygenase activity	0.688	0.002
Carotenoids versus proteinase activity	0.639	0.004
